# Correction: A protocol for modeling the factors influencing the deployment of the COVID-19 vaccine across African countries

**DOI:** 10.1371/journal.pone.0336507

**Published:** 2025-11-10

**Authors:** Obidimma Ezezika, Tiana Stephanie Kotsaftis, Edina Amponsah-Dacosta, Suleyman Demi, Eric Omori Omwenga, Samuel Mong’are, Trust Zaranyika, Oluwaseun Ariyo, Kandala Ngianga-Bakwin, Edward Kwabena Ameyaw

The Acknowledgement section is not included. The Acknowledgement section is as follows: Support from Western Data Solution Services (WDSS) at Western University is gratefully acknowledged for contributions to the development, refinement, and finalization of the model. Alanna Marson is acknowledged for developing the search strings used in the preliminary review, which informed the identification of potential variables included in the modeling. Appreciation is extended to Addy Lagunju for support during the finalization phase of the model, including assistance with data verification and documentation. Selina Quibrantar is acknowledged for her effective coordination of key project activities that directly contributed to the refinement of the model.
