# Supplementary material for: A protocol for modeling the factors influencing the deployment of the COVID-19 vaccine across African countries
Source: PLoS One. 2024 Nov 7;19(11):e0311800. doi: 10.1371/journal.pone.0311800 (PMC11542824; doi:10.1371/journal.pone.0311800)
Supplement: Attachment — Submitted filename: Commenters Response Regression Protocol July 26 TK_OE.docx [file pone.0311800.s001.docx]

| Comment | Response |
| --- | --- |
| **Reviewer 1** | |
| Authors apply the model to assess the influence of indicators on vaccination rates, which is very interesting. But, authors need to enhance the reliability of the method and the result need to There are the following points that need to interpret or supplement.  1.How to determine the candidate factor.  2. How to exclude pseudo-correlation.  3. Please interpret why to adopt linear regression model and add the describe of the model and the research step of the stepwise regression approach. | We will be going with a reduced model to enhance the reliability of our model, which we have delineated in the revised manuscript. We have included how the candidate factors are being determined through a review of the literature. In addition, we have included a section on the model evaluation including limitations and strengths:    *“To create a reduced model based on the correlation values, we aim to eliminate multicollinearity (high correlations) between predictors, to make our model more robust and interpretable. We will identify pairs of predictors with high absolute correlation values (e.g., > 0.7 or < -0.7) as these pairs indicate multicollinearity. We would then evaluate the adjusted R^2 values as a higher value indicates that the model is explaining a significant portion of the variance in the dependent variable, while also accounting for the number of predictors and avoiding overfitting. Once this is done, an ANOVA test will be run to identify significant differences in the models and see which reduced model significantly improves or worsens the model fit. For each pair of highly correlated predictors, we will decide which one to keep based on domain knowledge. We will select the reduced model that aligns with domain knowledge, has a higher adjusted R^2 values, and performs best on the ANOVA test. To ensure model accuracy, we are conducting a comprehensive diagnostic analysis. We are using the plot() function to generate a set of four diagnostic plots. These plots include a residuals vs. fitted values plot to check for homoscedasticity and linearity assumptions, a Q-Q plot to assess normality of residuals, a scale-location plot to check for homogeneity of variance, and a residuals vs. leverage plot to identify influential cases (Wood, 2017). We will also get a summary of the model’s performance across the five folds, including metrics such as RMSE and R-squared to assess for overfitting. This will help us understand how well the model generalizes to new data.”* |
| **Reviewer 2** | |
| 1. There is a wide array of previous literature that has considered regression analyses in relation to vaccination effectiveness and impact on the total number of cases of COVID-19. Can we add literature to introduce the situation of other regions and countries, as Asian countries and the US are mentioned in the article? | Thank you for your comment. We have addressed describing the use of regression analysis in relation to COVID-19 vaccination by adding four additional examples of areas not previously specified including Europe, the Middle East, Latin America and the Caribbean along with regions previously included, i.e, the US and Asia.  *In the European context, researchers utilized logistic regression models of vaccine hesitancy to determine what accounts for the variation in COVID-19 vaccine hesitancy across the continent (14). Regression models were also used in determining the hospitalization risk associated with COVID-19 vaccines along with their effectiveness at reducing the rate of hospital admissions in the United Arab Emirates (15). Lastly, in a study across 13 Latin American and Caribbean countries, regression analysis was one of the methods used to determine the prevalence of the intention to be vaccinated against COVID-19 and associated factors (16)*. |
| 2. There is very little introduction about the data analysis section. Can you provide a detailed introduction? | We have expanded the introduction in the data analysis section, which now reads as follows:  *We are inputting the independent variables (doses supplied, doses secured, doses administered, doses wasted, population size, HAQ index, GDP per capita, and World Bank income) and the dependent variable (COVID-19 vaccination rate) into a log format Excel sheet aligned according to the assigned country. To maintain consistency, we are standardizing the units with values in per capita being adjusted to the appropriate population size (Magezi, 2015). We are utilizing a reduced model and specifying it in an R data frame. The upstream independent factors stated previously, are being set as predictors in the model. These predictors will estimate the average population-level effect of each factor on the vaccination rate.* |
| 3. Can the conclusion drawn from the study of various African countries be compared with the results of other countries and regions? | Thank you for your comment. We have addressed the conclusions that are relevant to regions other than Africa by highlighting the fact that conclusions drawn from specific African countries and Africa as a continent and present as a country/continent-specific situation. However, data associated with the various factors will be available and can therefore be utilized by other countries or areas with similar profiles. Additionally, we have highlighted that results can be compared if additional tests are completed for regions outside of Africa.  *Conducting this analysis will allow for strong upstream predictors to be highlighted which may be relevant to other regions. Countries with similar profiles may be able to utilize conclusions drawn to help guide efforts but relevance would need to be tested and confirmed with subsequent analysis. To elaborate, other low-income countries with similar factors may be able to use conclusions drawn if a country in Africa has similar scores on the variety of factors. Additional tests and replication of this study utilizing a different region is plausible but would involve further analysis.* |
| 4. Does the conclusion about African countries obtained in this article have reference value for vaccination efforts in other countries? | Thank you for your comment. We have addressed conclusions obtained having reference value for vaccination efforts in other countries by adding that Africa is a unique situation as it was extremely far from the WHO’s vaccination readiness benchmark and the disparities across the continent highlighted.  *Although other low-income countries and continents face or have previously faced COVID-19 vaccination concerns, Africa presents itself as a unique case. As highlighted above, the range of vaccination varies greatly along with their readiness for a COVID-19 vaccination program falling well below WHO’s benchmark of 80% with 33% readiness (3).*  Additionally, in the discussion of our original manuscript, we highlighted that results may be applicable to vaccination efforts not only in other countries but also the rollout of other biomedical interventions.  *Recent reflections on COVID-19 vaccine rollout can provide crucial insights for future pandemic preparedness and other biomedical technologies. Further, the study’s potential reach and significance extend beyond geographical boundaries, and this study will have significant and far-reaching implications for overall pandemic preparedness and response against existing and emerging pathogens, including the rollout of other biomedical interventions such as antiviral therapies and rapid diagnosis tests.* |
| 5. Overfitting and underfitting are also plausible limitations that linear regression is susceptible to. How was it specifically addressed in the paper? | We have included several limitations and how they will be overcome including multicollinearity and overfitting. These are included in the new section on “Model Evaluation, Strengths and Limitations” |
| 6. Please use shorter sentences in the abstract section. | Thank you for your comment, the abstract has been proofread and sentences have been shortened to be more concise. |
| 7. Please write in a standardized manner. For example, there is no punctuation at the end of the Hypotheses section. | The manuscript has been proofread and additional edits done to resolve any typographical errors. |
